# Molecular characterization and genetic diversity analysis in Indian mustard (*Brassica juncea* L. Czern & Coss.) varieties using SSR markers

**DOI:** 10.1371/journal.pone.0272914

**Published:** 2022-08-26

**Authors:** K. H. Singh, Lal Singh, Nehanjali Parmar, Sunil Kumar, J. Nanjundan, Guman Singh, Ajay Kumar Thakur

**Affiliations:** 1 ICAR-Directorate of Rapeseed-Mustard Research, Bharatpur, Rajasthan, India; 2 ICAR-Indian Agricultural Research Institute-Regional Station, Wellington Tamilnadu, India; Chungnam National University, REPUBLIC OF KOREA

## Abstract

In this study, we evaluated genetic diversity in a panel of 87 Indian mustard varieties using 200 genomic-SSR markers. A total of 189 SSRs resulted into positive amplification with 174 (92.06%) SSRs generating polymorphic products and 15 (7.94%) SSRs producing monomorphic amplicons. A total of 552 alleles were obtained and allele number varied from 2–6 with an average number of 3.17 alleles per SSR marker. The major allele frequency ranged from 0.29 (ENA23) to 0.92 (BrgMS841) with an average value of 0.58 per SSR locus. The polymorphic information content (PIC) value ranged from 0.10 (BrgMS841) to 0.68 (BrgMS519) with 0.39 as mean PIC value. The gene diversity per locus ranged from 0.13 (BrgMS841) to 0.72 (ENA23 & BrgMS519) with a mean value of 0.48 per SSR primer pair. Both Unweighted Neighbor Joining-based dendrogram and population structure analysis divided all the 87 varieties into two major groups/subpopulations. Analysis of molecular variance (AMOVA) inferred the presence of more genetic variation (98%) among individuals than among groups (2%). A total of 31 SSRs produced 36 unique alleles for 27 varieties which will serve as unique DNA-fingerprints for the identification and legal protection of these varieties. Further, the results obtained provided a deeper insight into the genetic structure of Indian mustard varieties in India and will assist in formulating future breeding strategies aimed at Indian mustard genetic improvement.

## Introduction

Indian mustard (*Brassica juncea* L. Czern & Coss.) is an economically important oilseed crop of Rapeseed-Mustard group, belonging to family *Brassicaceae* with a physical genome size of 922 Mb [1]. It is an amphidiploid crop (AABB, 2n = 36), which evolved by natural hybridization between two primary diploids–*B*. *rapa* (AA, 2n = 20) and *B*. *nigra* (BB, 2n = 18), followed by subsequent chromosomal duplication in nature [2]. Presently, it is being cultivated in Canada, some European countries, Russia, Australia, China, Pakistan, India and Bangladesh. In India, it is being predominantly cultivated over >85% of the total RM acreage covering the areas of Rajasthan, UP, MP, Haryana, Gujarat, some of the southern states including Andhra Pradesh and Karnataka, and in some of the north-eastern states including Arunachal Pradesh, Meghalaya and Assam in a total acreage of about 6.2 million ha. Mustard oil is rich in monounsaturated and polyunsaturated fatty acids, has significant amount of omega-3 fatty acids and contains very less amount of saturated fatty acids, which make it quite useful from health point of view. Besides being used as cooking oil for edible purpose, its oil has found numerous other applications including use for body massage, in manufacturing paints and varnishes, and has the potential to be used as biofuel. Its seedmeal is very rich in proteins and provides an excellent feed for poultry animals [3]. However, the production and productivity of Indian mustard is severely affected by various biotic and abiotic stress constraints. The productivity of Indian mustard in India hovers around 1400 Kg/ha, which is abysmally very low. On the other hand, due to the increasing population and changing consumers’ preferences towards mustard oil, its demand and per capita consumption has been escalating day by day. India imports around 57% of edible oil from other countries, hence involving a large exchequer of revenue of around Rs 73,500 crores per annum [4]. There are projections that our edible oil demand will reach upto 34 million tonnes by 2025, out of which 14 million tonnes has to be contributed by Indian mustard alone. This has exerted a tremendous pressure on developing high yielding varieties, which can perform well under the changing regime of climate through conventional plant breeding and modern biotechnological interventions. For formulating an effective breeding program for Indian mustard improvement, estimation of genetic diversity is of utmost importance to identify and select the most divergent genotypes as donors/parents.

In India, no comprehensive effort has been attempted so far to evaluate genetic diversity inherent in Indian mustard varieties except for few scattered reports [5–8]. As International Union for Protection of New Varieties of Plants (UPOV) has made it mandatory to have DNA fingerprinting profile for legal protection of a plant variety [9], there is an imminent need to establish a unique system for identification of promising cultivars of Indian mustard for their protection under the current IPR regime. Further, many cultivars exhibit almost similar phenotypes/overlapping traits, so it becomes a herculean task to identify them on the basis of morphological characteristics [7], which entails the need of developing unique DNA fingerprints for their accurate identification.

In recent years, with the faster development in molecular biology techniques and the availability of genomic resources, DNA-based molecular markers are being increasingly employed for rapid identification of cultivars, their fingerprinting and diversity analysis. Among various type of DNA-based markers, SSRs have become number one choice of plant breeders and biotechnologists for genetic diversity evaluation and varietal characterization because of their high abundance, reproducibility, simplicity, co-dominant inheritance, higher polymorphism rate and wider genome coverage [10]. SSR markers assist in identification of duplicates, synonyms and homonyms among crop varieties and are being utilized as the most successful and promising molecular marker system for developing plant DNA-fingerprint database [11], genetic diversity analysis [12], marker- assisted breeding [13] and varietal purity identification [14].

All the Indian mustard varieties have a narrow genetic base [15], hence they exhibit very low level of polymorphism which hampers genetic improvement in this crop. In this context, the use of large-scale polymorphic SSR markers distributed on all the linkage groups of Indian mustard would greatly facilitate molecular characterization for the correct identification of Indian mustard cultivars for the protection of plant breeder’s rights and genetic diversity analysis. In the present study, we have used an already identified and validated set of polymorphic SSR markers [16] for genetic diversity and population structure analysis and developing molecular tags/DNA fingerprints for Indian mustard cultivars.

## Materials and methods

### Plant material

Selfed, pure seeds of eighty-seven Indian mustard varieties were taken from DUS division of ICAR-DRMR, Bharatpur, Rajasthan, which comprised of the plant material in this study. The detailed information of these varieties along with their developing center, pedigree and release year is given in Table 1. SSR-genotyping work had been carried out in Molecular Biology Laboratory of ICAR-DRMR, Bharatpur, Rajasthan, India.

**Table 1:** List of *B*. *juncea* varieties used in the present investigation and their pedigree/description.

S. No.	Name of the Variety	Developing Centre	Pedigree	Year of release
1	ACN Satabdi	College of Agriculture, Nagpur, Maharashtra	Seeta x RW 351	2005
2	Arawali	ARS, Navgaon, RAU Bikaner	Krishna x RS 50	1998
3	Ashirwad	CSAUA & T, Kanpur, Uttar Pradesh	Krishna x Vardan	2005
4	Basanti	CSAUA & T, Kanpur, Uttar Pradesh	Varuna x RK 1	2000
5	Bhagirathi	PORS, Berhampore, West Bengal	Varuna x Appressed mutant (APM)	1984
6	BR-40	Govt. of Bihar	Pureline selection from local germplasm	1960
7	CS-52	CSSRI, Karnal, Haryana	Selection from DIRA 343	1997
8	CS-54	CSSRI, Karnal, Haryana	B 380 x NDR 8603	2003
9	Durgamini	Deptt. of Agriculture, Govt. of Rajasthan	Selection from the material collected from Sriganganagar, Rajasthan	1968
10	Geeta	RRS Bawal, Haryana	Spontaneous mutant of cultivar RH 30	2002
11	GM-1	SDAU, SK Nagar, Gujarat	MR 71-3-2 x TM 4	1989
12	GM-2	SDAU, SK Nagar, Gujarat	Selection from material collected from Vendancha, Gujarat	1996
13	GM- 3	SDAU, SK Nagar, Gujarat	RSK 78 x Varuna	2004
14	Pusa Jagannath	IARI, New Delhi	Varuna x Synthetic *juncea*	1998
15	JM- 1	ZARS, Morena, MP	Pusa Bold x L 6	1999
16	JM-2	ZARS, Morena, MP	Varuna x L 4	2004
17	JM- 3	ZARS, Morena, MP	Varuna x YRT 3	2004
18	Kanti	CSAUA & T, Kanpur, Uttar Pradesh	Selection from germplasm collected from Kanpur Dehat	2002
19	Pusa Karishma	IARI, New Delhi	Pusa Basanti x ZEM 1	2004
20	Kranti	GBPUA & T, Pantnagar, Uttarakhand	Selection from Varuna	1982
21	Krishna	GBPUA & T, Pantnagar, Uttarakhand	Selection from Varuna	1983
22	Laxmi	CCSHAU, Hisar, Haryana	PR 15 x RH 30A	1996
23	Prakash	CCSHAU, Hisar, Haryana	RL 18 x T 9	1974
24	Maya	CSAUA & T, Kanpur, Uttar Pradesh	Varuna x KRV 11	2002
25	Navgold	ARS, Navgaon, RAU Bikaner	Bio 902 x BM 185–11	2005
26	NDRE- 4	NDUA & T, Faizabad, Uttar Pradesh	TM 9 x Seeta	1999
27	Narendra Rai	NDUA & T, Faizabad, Uttar Pradesh	Selection from material collected from Atwa, U.P.	1990
28	NRCDR- 2	DRMR, Bharatpur, Rajasthan	MDOC 43 x NBPGR 36	2006
29	Pusa Agrani	IARI, New Delhi	Early maturing *Brassica juncea* x Synthetic amphidiploid (*Brassica campestris* var. toria x *Brassica nigra*)	1997
30	Pusa Bahar	IARI, New Delhi	(Pusa Rai 28 x Varuna) x (Pusa Rai 30 x T 6342)	1989
31	Pusa Bold	IARI, New Delhi	Varuna x BIC 1780	1984
32	Pusa Jaikisan	NRCPB, IARI, New Delhi	Somaclone of Varuna	1993
33	Pusa Mahak	IARI, New Delhi	Pusa Bold x Glossy mutant	2004
34	PBR- 91	RRS, Bathinda, Punjab	(RLM 511 x PR 18) x CM 1	1994
35	PBR 97	RRS, Bathinda, Punjab	(DIR 202 x PR 34 x V 3) x (RLM 619 x Varuna)	1996
36	PBR- 210	RRS Bathinda, Punjab	Not available	2003
37	Rajat	CCSHAU, Hisar, Haryana	Selection from Kutch germplasm line JMG 36–3	1993
38	PM- 67	Deptt. of Agriculture, Govt. of Gujarat	Selection from local material of Gujarat	1967
39	RB-50	RRS, Bawal, Haryana	Laxmi x RH 9617	2008
40	RCC-4	SAREC, Kangra, HP	Selection from a multiple cross involving Varuna, Pusa Bold, Pusa Bold 75–2, Pant 18, RH 30, RLM 171, RH 7301 and RLM 504	1996
41	RGN-13	ARS, Sriganganagar, Rajasthan	RH 30 x Varuna	2002
42	RGN-48	ARS, Sriganganagar, Rajasthan	RSM 204 x B 75	2004
43	RGN-73	ARS, Sriganganagar, Rajasthan	RGN 8 x Pusa Bold	2006
44	RH-30	CCSHAU, Hisar, Haryana	P 26 x 3–1	1983
45	RH-781	CCSHAU, Hisar, Haryana	(RL18 x P26/3-1) x RL 18	1990
46	RH-819	CCSHAU, Hisar, Haryana	Prakash x Bulk pollen	1990
47	RL-1359	PAU, Ludhiana, Punjab	RLM 514 x Varuna	1987
48	RLM-619	PAU, Ludhiana, Punjab	Gamma rays induced mutant of RL 18	1983
49	Rohini	CSAUA & T, Kanpur, Uttar Pradesh	Selection from natural population of Varuna	1985
50	RRN-505	ARS, Navgaon, Rajasthan	Pusa Bhusan x ABRNT 1	2005
51	Sarama	PORS, Berhampore, West Bengal	Varuna x B5	1984
52	Shivalik	Mahyco, Jalana, Maharashtra	Not available	2002
53	Shivani	BAU, Ranchi, Jharkhand	Selection from local germplasm	2003
54	RRN-573	ARS, Navgaon, Rajasthan	HUM-9504 (*B*. *juncea*) X GSH 1 (*B*. *napus*)	2013
55	Sanjucta Asech	PORS, Berhampore, West Bengal	TM4 x RK2	1989
56	TM-2	BARC, Trombay, Mumbai	Gamma rays induced mutant of RL 9	1987
57	Urvashi	CSAUA & T, Kanpur, Uttar Pradesh	Varuna x Kranti	1999
58	Vaibhav	CSAUA & T, Kanpur, Uttar Pradesh	Derived through biparental mating involving Varuna, Keshari, CSU 10 and IB1775, IB1786 and IB 1866	1985
59	Varuna	CSAUA & T, Kanpur, Uttar Pradesh	Selection from Varanasi Local	1975
60	Vasundhara	CCSHAU, Hisar, Haryana	RH 839 x RH 30	2002
61	Vardan	CSAUA & T, Kanpur, Uttar Pradesh	Derived through biparental mating involving Varuna, Keshari, CSU 10 and IB 1775, IB 1786, IB 1866	1985
62	Pusa Mustard-27	IARI, New Delhi	Derived from the cross [(Divya x Pusa Bold) x (PR 666EPS) x PR 704EPS-2 x B85)]	2011
63	HYT-33	IARI, New Delhi	Pusa Bold x Pusa Barani	Not available
64	Pusa Mustard- 21	IARI, New Delhi	Pusa Bold x ZEM 2	2006
65	Pusa Mustard- 22	IARI, New Delhi	Pusa Barani x ZEM 2	2007
66	Pusa Mustard- 24	IARI, New Delhi	(Pusa Bold x LEB 15) x LES 29	2007
67	Pusa Mustard- 29	IARI, New Delhi	(ZEM-2 x Pusa Barani) x EC-287711	2013
68	Pusa Vijay	IARI, New Delhi	Synthetic *Brassica juncea* x VSL 5	2006
69	Pusa Mustard- 25	IARI, New Delhi	SEJ 8 x Pusa Jagannath	2009
70	Pusa Mustard- 26	IARI, New Delhi	VEJ Open x Pusa Agrani	2010
71	Pusa Mustard- 28	IARI, New Delhi	SEJ 8 x Pusa Jagannath	2011
72	Pusa Tarak	IARI, New Delhi	Agra Local x Poorbi Raya	2006
73	RGN-236	ARS, Sriganganagar, Rajasthan	SBG-00-01 x Laxmi	2013
74	RGN-229	ARS, Sriganganagar, Rajasthan	HEB-3 x Laxmi	2013
75	RH-406	CCSHAU, Hisar, Haryana	RH 9608 X RH 30	2013
76	Sitara Singar	Bharatpur, Rajasthan (Farmer’s variety)	Selection from local germplasm of Bharatpur	Not available
77	Giriraj	DRMR, Bharatpur, Rajasthan	HB 9908 x HB 9916	2013
78	Swaran Jyoti	CCSHAU, Hisar, Haryana	Selection from germplasm line RC 1670	2002
79	RH-749	CCSHAU, Hisar, Haryana	RH 781 x RH 9617	2012
80	RGN-298	ARS, Sriganganagar, Rajasthan	RGN 96 x Pusa Bold	2015
81	RVM-2	RVSKVV, Jabalpur, MP	Selection from Chambal growing region	2013
82	RLC-2	PAU, Ludhiana, Punjab	QM 4 x Pusa Bold	2011
83	Pusa Mustard- 30	IARI, New Delhi	Bio-902 x ZEM-1	2013
84	DRMR-601	DRMR, Bharatpur, Rajasthan	NBPGR 272 x RK 9903	2010
85	RH-119	CCSHAU, Hisar, Haryana	Pusa Bold x Rajat	2009
86	DRMR-150-35	DRMR, Bharatpur, Rajasthan	RH 819 x Pusa Bold	2015
87	NRCHB-101	DRMR, Bharatpur, Rajasthan	BL 4 x Pusa Bold	2008

### DNA extraction and purification

DNA from pooled fresh and young leaves of five plants per genotype was extracted and purified using the already high stringency protocol in our laboratory [17]. The concentration of purified DNA was examined by running on 0.8% agarose gel electrophoresis along with a lambda DNA ladder.

### SSR primers and polymerase chain reaction (PCR) amplification

A panel of 200 genomic-SSRs that covers all eighteen linkage groups of Indian mustard [16] were chosen for genotyping of Indian mustard varieties. Polymerase chain reactions were run following the high stringency protocol already standardized in our laboratory [18]. PCR amplicons were resolved on 3.5% Super Fine Resolution (SFR) agarose (Amresco, USA) gel along with 50 bp DNA ladder as a benchmark on both sides of the gel and analyzed in a gel documentation system (Syngene, UK).

### Data analysis

PCR amplicons of different sizes were considered as different alleles. An allelic size data matrix was prepared and subjected to PowerMarker v.3.25 [19] for calculation of major allele frequency (MAF), polymorphism information content (PIC) value and gene diversity. Variety wise allelic composition of SSR markers was prepared from the score sheet and unique alleles (particular allele appearing only in one variety) were identified as DNA fingerprint for a particular variety. UNJ-dendrogram was constructed using Darwin 5.0 software [20] to decipher genetic relationship among different varieties used.

### Population structure analysis and AMOVA

Analysis of population structure of Indian mustard cultivars was carried out by STRUCTURE v2.3.4 software using admixture model [21]. For reach value of K (from 1–9), five independent runs were carried out with a burn-in period of 1,000,000 followed by 100,000 Markov Chain Monte Carlo (MCMC) replications. STRUCTURE HARVESTER v.6.92 [22] was used to determine the optimum K value by log probability of data [ln P(D)]. Indian mustard genotypes were classified into two classes; pure–the genotypes with ≥80% affiliation probabilities and admixture–with ≤80% affiliation probabilities. GenAlEx6.5 software [23] was used for analysis of molecular variance (AMOVA).

## Results

### Allelic diversity and genetic inter-relationship analysis

**Fig 1:**
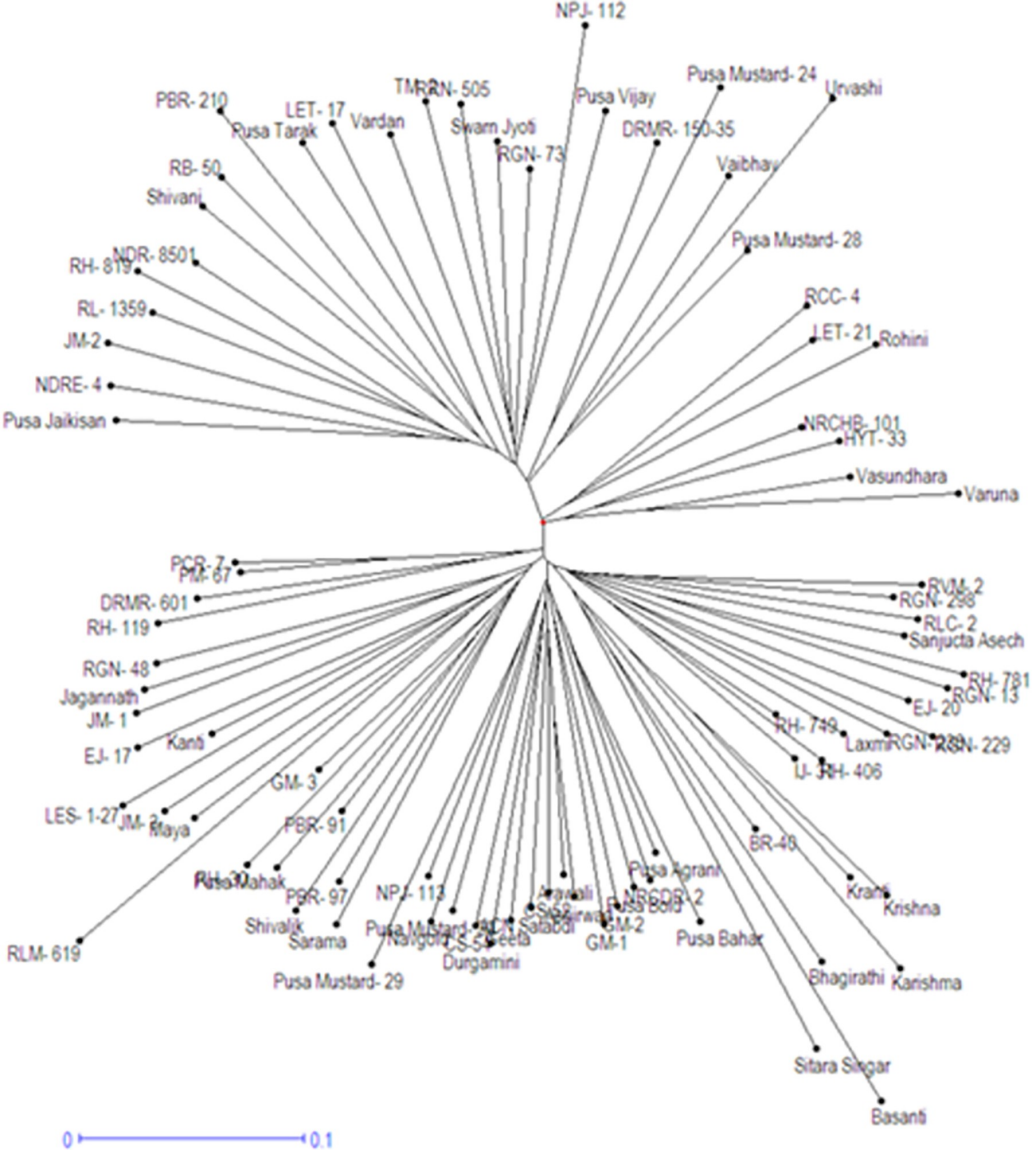
UNJ-dendrogram showing genetic relationship among 87 Indian mustard varieties using 174 SSR markers.

Out of 200 SSRs evaluated, 189 SSR markers produced clear and scorable bands, while remaining 11 exhibited no amplification at all. Among the amplified SSR markers, 174 (92.06%) SSRs amplified polymorphic products, whereas 15 (7.94%) SSRs resulted into monomorphic amplicons. Various allelic diversity parameters of SSR markers used in this study including number of alleles, major allele frequency (MAF), PIC value and gene diversity are presented in Table 2. A total of 552 alleles were obtained and the allele number ranged from 2–6 with an average number of 3.17 alleles per SSR locus. The major allele frequency varied from 0.29 (ENA23) to 0.92 (BrgMS841) with a mean value of 0.58 per SSR marker. PIC value defines the discriminatory power of a marker and is the representative of allelic diversity and frequency among the genotypes. The PIC value was in the range of 0.10 (BrgMS841) to 0.68 (BrgMS519) with 0.39 as mean PIC value. The gene diversity per locus ranged from 0.13 (BrgMS841) to 0.72 (ENA23 & BrgMS519) with a mean value of 0.48 per SSR primer pair. SSR marker polymorphism level is generally measured in terms of PIC values. The discriminatory power of SSR marker can be defined as high for PIC values >0.50, moderate for PIC values in the range of 0.25 to 0.50 and low for PIC values <0.25 [24]. In the present study, 19 (10.92%) SSR markers were highly polymorphic and informative having PIC values >0.50, 154 (88.51%) SSRs were moderately polymorphic (PIC values in the range of 0.25–0.50) and one (0.57%) SSR marker exhibited low degree of informativeness and polymorphic potential (with PIC values <0.25). A total of 44 SSR markers resulted into PIC values greater than the mean PIC value, which infers that these SSRs can be used for various trait mapping studies in *B*. *juncea*. UNJ-based grouping method using euclidean distance matrix based upon SSR allelic data grouped all the 87 varieties into two major clusters (Fig 1). Fifty-seven varieties were grouped into cluster I and remaining 30 were grouped into cluster II.

**Table 2:** Various allelic diversity parameters of polymorphic SSR markers used for genotyping of Indian mustard varieties.

S. No.	Marker ID	Chromosome No./Linkage group	No. of alleles	Major allele frequency	PIC value	Gene diversity
1	BRMS-002	-	3	0.34	0.59	0.66
2	BRMS-003	-	3	0.38	0.56	0.63
3	BRMS-005	-	3	0.65	0.35	0.45
4	BRMS-006	B3	4	0.68	0.37	0.44
5	BRMS-011	-	5	0.77	0.30	0.35
6	BRMS-015	-	3	0.60	0.39	0.49
7	BRMS-017	-	5	0.77	0.30	0.35
8	BRMS-029	-	2	0.51	0.37	0.49
9	BRMS-033	-	3	0.63	0.37	0.46
10	BRMS-040	-	6	0.64	0.46	0.50
11	Ra1-F06	A6	2	0.50	0.38	0.50
12	Ra2-A01	A1	5	0.65	0.43	0.49
13	Ra2-A11	A1	4	0.67	0.38	0.45
14	Ra2-C09	A3	5	0.64	0.45	0.50
15	Ra2-D04	A4	3	0.44	0.51	0.59
16	Ra2-E04	A5	6	0.74	0.35	0.39
17	Ra2-E11	A5	6	0.66	0.45	0.50
18	Ra2-E12	A5	3	0.35	0.58	0.65
19	Ra2-F11	A6	3	0.38	0.55	0.63
20	Ra2-G05	A7	6	0.68	0.43	0.48
21	Ra3-H09	A8	4	0.72	0.33	0.40
22	BrgMS10	A7	5	0.54	0.54	0.60
23	BrgMS13	A8	2	0.51	0.37	0.49
24	BrgMS33	A6	4	0.67	0.38	0.45
25	BrgMS54	A7	3	0.65	0.36	0.45
26	BrgMS59	A7	5	0.77	0.30	0.35
27	BrgMS64	A8	3	0.54	0.44	0.52
28	BrgMS66	A8	5	0.59	0.50	0.56
29	BrgMS68	A5	2	0.51	0.37	0.49
30	BrgMS75	A2	2	0.51	0.37	0.49
31	BrgMS89	A2	4	0.73	0.32	0.39
32	BrgMS90	A6	2	0.50	0.38	0.50
33	BrgMS139	A7	3	0.56	0.42	0.51
34	BrgMS147	A5	4	0.71	0.34	0.41
35	BrgMS162	A4	3	0.64	0.37	0.46
36	BrgMS166	A5	6	0.72	0.38	0.42
37	BrgMS171	A7	3	0.53	0.45	0.53
38	BrgMS175	A1	3	0.67	0.34	0.44
39	BrgMS202	A7	2	0.52	0.36	0.48
40	BrgMS216	A10	2	0.52	0.36	0.48
41	BrgMS233	A4	4	0.69	0.37	0.44
42	BrgMS236	A2	2	0.52	0.36	0.48
43	BrgMS247	A4	4	0.75	0.30	0.37
44	BrgMS268	A2	2	0.51	0.37	0.49
45	BrgMS286	A6	3	0.57	0.42	0.51
46	BrgMS301	A2	5	0.78	0.29	0.34
47	BrgMS334	A10	2	0.53	0.35	0.47
48	BrgMS338	A7	4	0.66	0.38	0.45
49	BrgMS344	A7	3	0.69	0.32	0.40
50	BrgMS347	A9	3	0.61	0.39	0.48
51	BrgMS352	A9	2	0.53	0.35	0.47
52	BrgMS360	A6	2	0.52	0.36	0.48
53	BrgMS372	A8	3	0.66	0.34	0.44
54	BrgMS377	A9	3	0.66	0.35	0.45
55	BrgMS388	A2	3	0.65	0.36	0.46
56	BrgMS389	A9	2	0.53	0.35	0.47
57	BrgMS397	A2	3	0.60	0.39	0.48
58	BrgMS399	A9	3	0.61	0.39	0.48
59	BrgMS409	A6	2	0.50	0.38	0.50
60	BrgMS412	A2	3	0.62	0.38	0.48
61	BrgMS414	A1	2	0.53	0.35	0.47
62	BrgMS421	A3	4	0.73	0.33	0.40
63	BrgMS422	A3	3	0.57	0.41	0.50
64	BrgMS426	A4	2	0.51	0.37	0.49
65	BrgMS430	A1	2	0.52	0.36	0.48
66	BrgMS433	A1	3	0.48	0.49	0.57
67	BrgMS455	A10	3	0.49	0.48	0.56
68	BrgMS457	A5	3	0.46	0.50	0.58
69	BrgMS465	A8	6	0.82	0.26	0.30
70	BrgMS519	A8	5	0.39	0.68	0.72
71	BrgMS521	A5	2	0.52	0.36	0.48
72	BrgMS565	A1	4	0.67	0.38	0.45
73	BrgMS566	A2	4	0.67	0.38	0.45
74	BrgMS570	A1	2	0.54	0.34	0.46
75	BrgMS590	A4	2	0.55	0.34	0.45
76	BrgMS595	A10	3	0.36	0.57	0.64
77	BrgMS635	A1	2	0.51	0.37	0.49
78	BrgMS638	A4	3	0.59	0.40	0.49
79	BrgMS643	A5	4	0.73	0.32	0.39
80	BrgMS684	A6	5	0.72	0.36	0.41
81	BrgMS688	A9	3	0.39	0.54	0.61
82	BrgMS691	A5	2	0.51	0.37	0.49
83	BrgMS713	A10	3	0.34	0.58	0.66
84	BrgMS738	A9	3	0.62	0.38	0.47
85	BrgMS746	A9	3	0.59	0.40	0.49
86	BrgMS751	A2	2	0.53	0.35	0.47
87	BrgMS776	A9	2	0.58	0.31	0.42
88	BrgMS778	A6	2	0.51	0.37	0.49
89	BrgMS780	A9	3	0.59	0.40	0.49
90	BrgMS782	A1	3	0.64	0.36	0.44
91	BrgMS787	A6	5	0.70	0.38	0.43
92	BrgMS794	A10	2	0.54	0.34	0.46
93	BrgMS799	A3	3	0.40	0.54	0.61
94	BrgMS801	A1	3	0.70	0.30	0.39
95	BrgMS825	A8	2	0.50	0.38	0.50
96	BrgMS841	A4	4	0.92	0.10	0.13
97	BrgMS961	A9	2	0.52	0.36	0.48
98	BrgMS1238	A1	3	0.34	0.58	0.66
99	BrgMS1466	A3	3	0.66	0.35	0.45
100	BrgMS1774	A5	3	0.65	0.36	0.45
101	BrgMS2996	A3	4	0.73	0.32	0.39
102	BrgMS3322	A9	3	0.33	0.59	0.67
103	BrgMS4497	A3	4	0.38	0.62	0.67
104	BrGMS4508	A7	2	0.55	0.34	0.45
105	BrgMS4509	A10	2	0.51	0.37	0.49
106	BrgMS4513	A10	2	0.53	0.35	0.47
107	BrgMS4514	A10	2	0.51	0.37	0.49
108	BrGMS4533	A8	4	0.72	0.33	0.39
109	BrGMS4536	A8	2	0.52	0.36	0.48
110	BrgMS4539	A2	4	0.43	0.59	0.66
111	BrgMS4543	A2	3	0.33	0.59	0.67
112	Ni2A01	-	2	0.51	0.37	0.49
113	Ni2A02	-	2	0.50	0.38	0.50
114	Ni2A08	-	4	0.73	0.32	0.39
115	Ni2A12	-	2	0.53	0.35	0.47
116	Ni2D10	-	2	0.54	0.34	0.46
117	Ni3C05	-	4	0.76	0.29	0.36
118	Ni3H07	-	3	0.67	0.34	0.43
119	Ni4C02	-	3	0.54	0.43	0.52
120	Ni4C06	-	3	0.63	0.37	0.46
121	Ni4C09	-	6	0.63	0.48	0.52
122	Ni4C11	-	3	0.36	0.57	0.65
123	Ni4F09	-	4	0.71	0.34	0.41
124	Ni4F11	-	5	0.79	0.27	0.32
125	SB0372	B4	4	0.79	0.25	0.31
126	SB1728	B8	3	0.63	0.37	0.45
127	SB1937	B7	2	0.50	0.38	0.50
128	SB2131	B4	3	0.66	0.34	0.44
129	SB2556	B5	3	0.68	0.33	0.42
130	SB3140	B5	2	0.50	0.38	0.50
131	SB3751	B8	2	0.51	0.37	0.49
132	SB3872	B5	2	0.53	0.35	0.47
133	SB4817	B2	2	0.51	0.37	0.49
134	SB5162	B8	4	0.71	0.34	0.40
135	SB05631	B1	2	0.53	0.35	0.47
136	SJ0338	B6	3	0.65	0.36	0.46
137	SJ0502	B6	2	0.51	0.37	0.49
138	SJ1505	B6	3	0.56	0.43	0.51
139	SJ1536	B7	3	0.64	0.37	0.46
140	SJ1668I	B8	2	0.53	0.35	0.47
141	SJ3640I	B6	3	0.67	0.34	0.43
142	SJ3838	B1	6	0.72	0.37	0.42
143	SJ3874I	B5	4	0.76	0.29	0.35
144	SJ6846	B2	4	0.70	0.35	0.42
145	SJ7104	B6	2	0.51	0.37	0.49
146	SJ8033	B4	4	0.72	0.34	0.41
147	SJ13133	B7	3	0.57	0.41	0.50
148	SJ34121	B8	3	0.62	0.38	0.48
149	SJ39119I	B7	3	0.65	0.36	0.46
150	nia-m066a	-	3	0.70	0.31	0.40
151	nia-m085a	-	4	0.72	0.33	0.40
152	nia-m091a	-	2	0.50	0.38	0.50
153	cnu_m584a	A8	4	0.74	0.31	0.38
154	cnu_m587a	-	3	0.67	0.34	0.43
155	cnu_m593a	-	3	0.46	0.49	0.57
156	cnu_m594a	-	3	0.65	0.36	0.45
157	cnu_m596a	-	2	0.51	0.37	0.49
158	cnu_m597a	-	5	0.72	0.34	0.39
159	cnu_m626a	A9	3	0.60	0.39	0.48
160	MB4	-	2	0.52	0.36	0.48
161	KBRH139B23	-	3	0.62	0.38	0.47
162	E129	-	2	0.51	0.37	0.49
163	EJU1	A9	3	0.38	0.56	0.63
164	EJU3	-	4	0.51	0.54	0.60
165	EJU4	-	3	0.36	0.57	0.65
166	EJU5	-	5	0.63	0.46	0.52
167	ENA19	-	2	0.51	0.37	0.49
168	ENA20	-	3	0.65	0.36	0.45
169	ENA21	-	2	0.51	0.37	0.49
170	ENA23	A2	4	0.29	0.67	0.72
171	MR52a	-	2	0.51	0.37	0.49
172	Ol10B07	-	4	0.76	0.29	0.34
173	Ol10B11	-	4	0.74	0.30	0.36
174	PW243	-	3	0.66	0.35	0.45
**Mean**			**3.17**	**0.58**	**0.39**	**0.48**

### Population structure analysis and AMOVA

**Fig 2:**
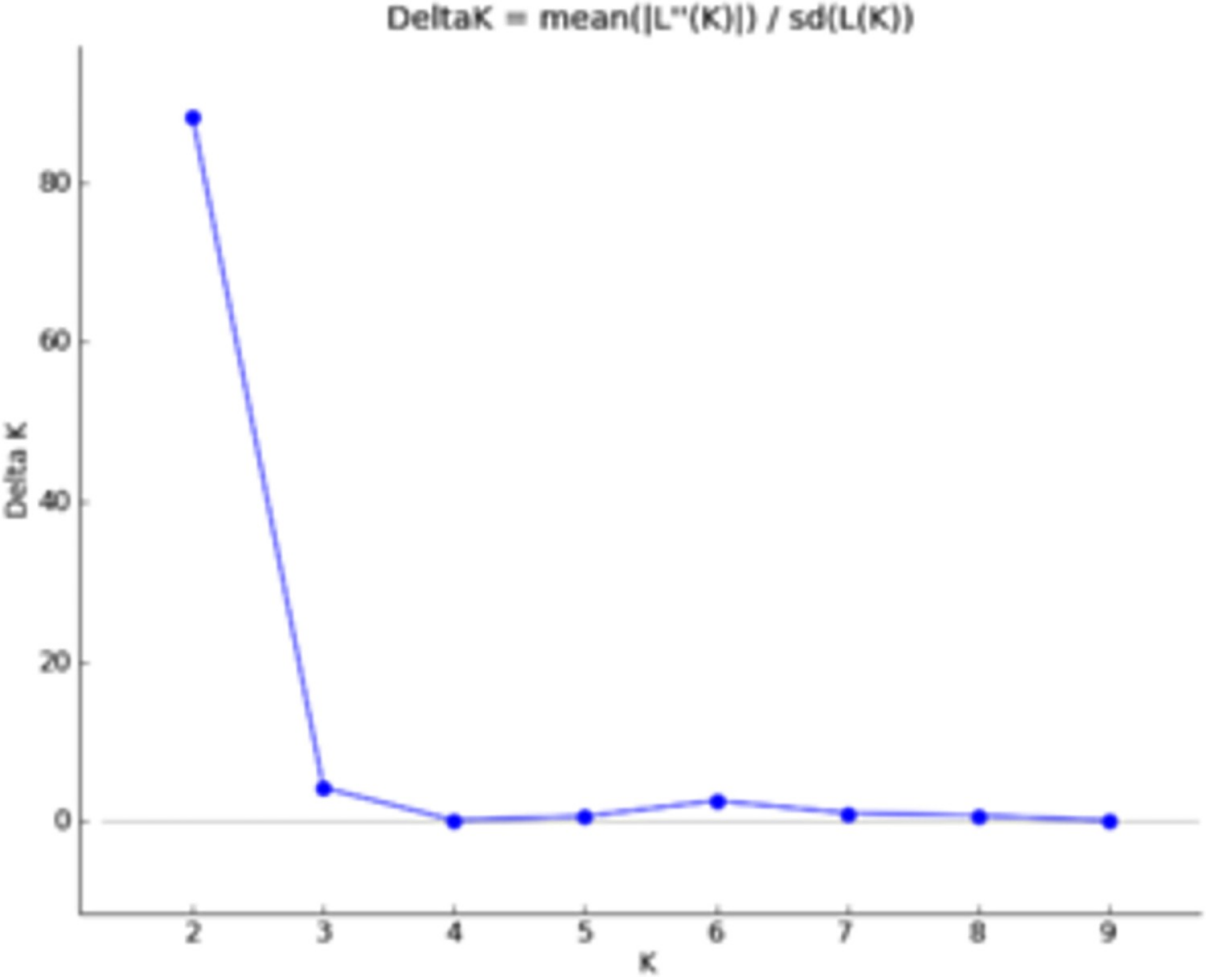
Population structure analysis using LnP(D) derived delta K for determining optimum number of subpopulations. The maximum of adhoc measure delta K determined by Structure Harvester was found to be K = 2, which inferred that the whole population can be divided into 2 subpopulations.

**Fig 3:**
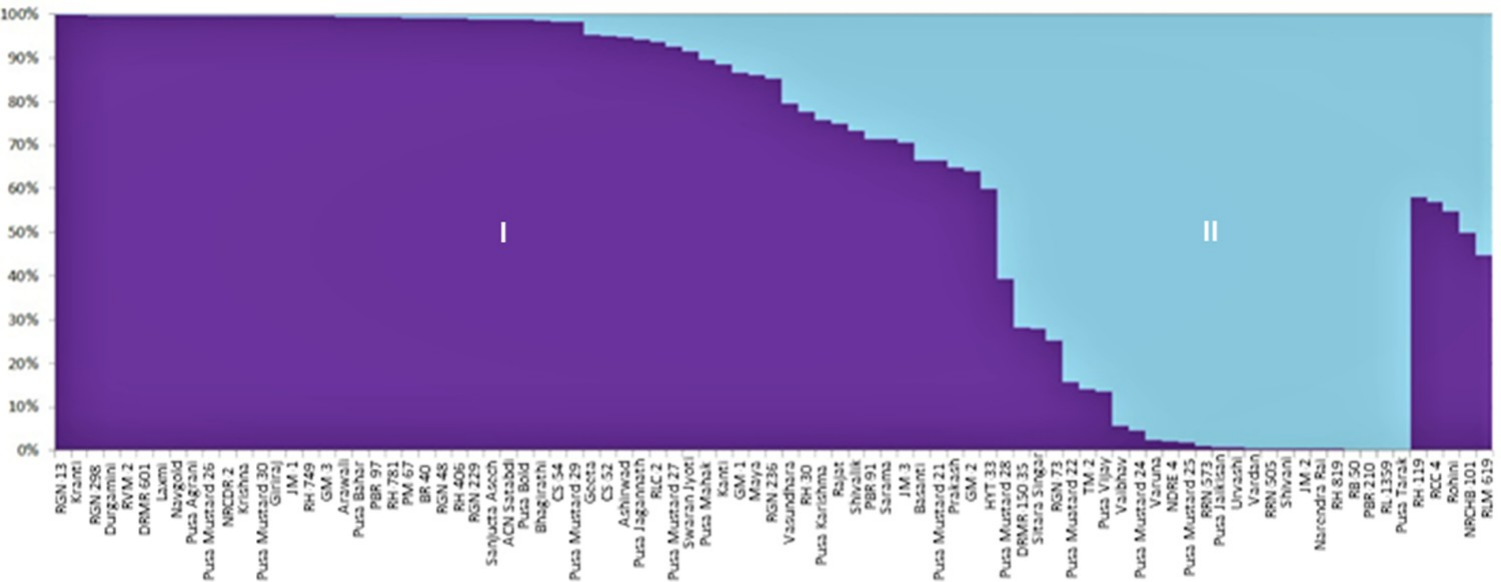
Population structure of 87 Indian mustard varieties on the basis of allelic data of 174 SSR markers.

Population structure analysis enhances the understanding about genetic relationship among various genotypes and also provides the basis for association mapping studies. Till now, no effort has been directed to study the population structure of Indian mustard cultivars. In the present study, the results based on K-value of 1–9 exhibited a sharp peak of delta K at K = 2 (Fig 2) inferring the presence of two subpopulations and all further interpretations were carried out according to this K value. The allelic variation patterns in the bar diagram (Fig 3) inferred the presence of large-scale admixtures which means such genotypes have mixed parentage belonging to dissimilar gene pools. Based on the probability criterion of membership of ≥0.80 for any cultivar to be pure, 61 genotypes were assigned to SP1, out of which 44 (50.57%) cultivars were pure lines, while 17 (19.54%) were admixtures; while in SP2, from a total of 26 genotypes, 21 (24.14%) were pure lines and 5 (5.75%) genotypes were of admixture type. The presence of admixtures indicates that natural outcrossing and cross-hybridization had been practiced in the past to develop these varieties.

**Fig 4:**
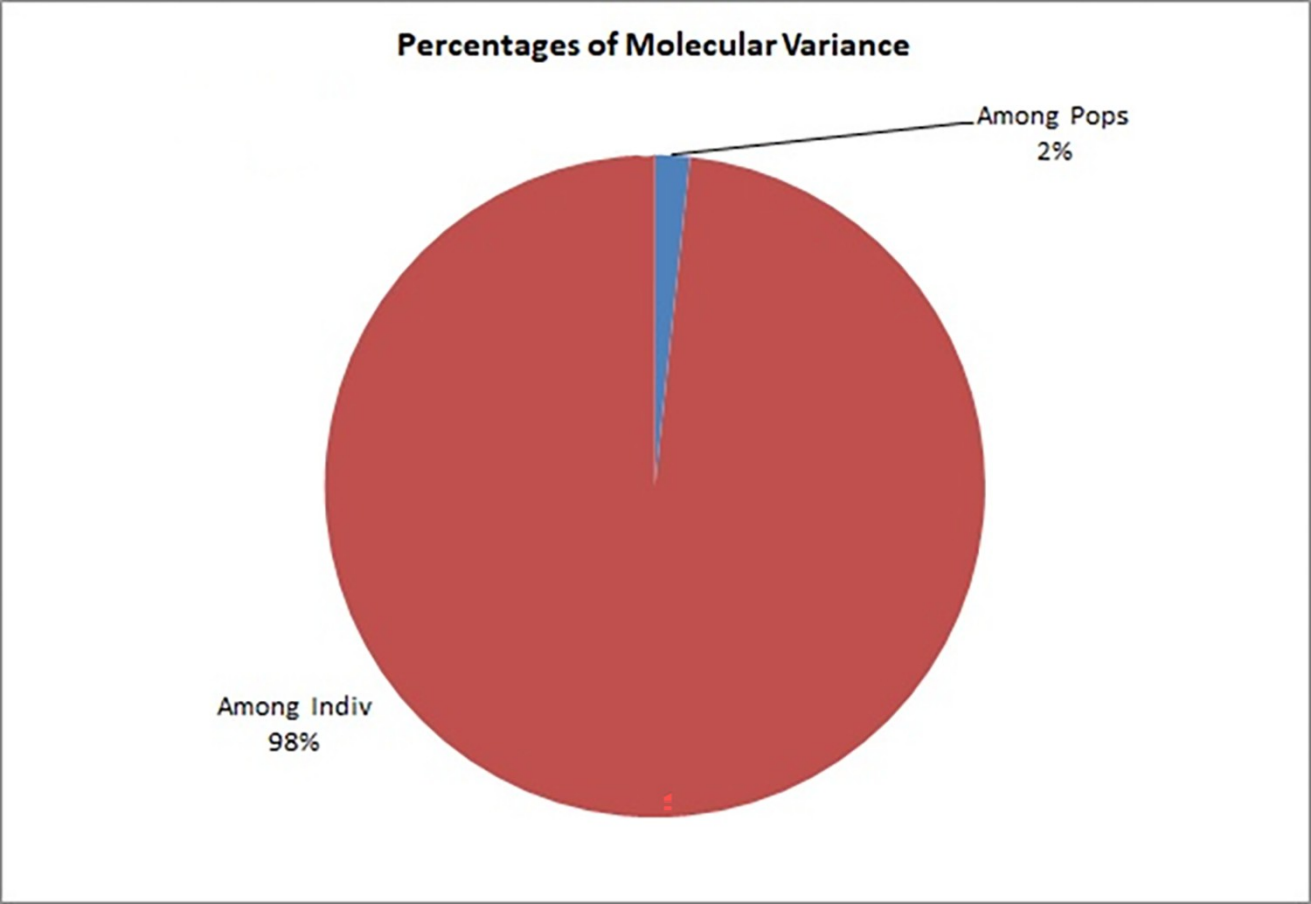
Analysis of molecular variance (AMOVA) of Indian mustard cultivars based on SSR data.

The subpopulations as obtained by population structure analysis were subjected to analysis of molecular variance (AMOVA) to quantify the percentage of variation among and within subpopulations. AMOVA explained that most of the genetic variation of this species resides among the cultivars which accounted for 98% of the total variation, while remaining 2% of the genetic variation was attributed to the between populations genetic variation (Fig 4, Table 3). We can draw the inference that the main genetic variations have originated from differences among the individuals and not from the different groups.

**Table 3:** Analysis of molecular variance (AMOVA) of Indian mustard cultivars based on SSR data.

Source of variation	df	SS	MSS	Est. Var.	%age
**Among populations**	2	277.08	138.54	0.88	2
**Among individuals**	84	8519.86	101.42	50.71	98
**Total**	86	8796.94		51.59	100

### Unique alleles/DNA fingerprint development

In the present study, SSR allele size matrix was analyzed to identify unique genotype-specific alleles (particular allele appearing only in one variety) to demarcate SSR-based DNA fingerprint for a particular variety. A total of 36 unique alleles were reported in the dataset with 31 SSR markers to distinguish 27 Indian mustard varieties (Table 4).

**Table 4:** Unique alleles/DNA fingerprint for selected Indian mustard varieties.

S.No.	SSR marker	Genotype/variety	Unique allele (bp)	S.No.	SSR marker	Genotype/variety	Unique allele (bp)
1	BrgMS1238	Basanti	270	16	SJ3874I	Pusa Mustard-24	170
2	BrgMS638	Pusa Mustard-24	270	17	SB0372	DRMR 150–35	235
3	cnu_m593a	Durgamini	200	18	SB2556	RH-749	225
4	Ra2-G05	EJ-20	170	19	BrgMS465	Bhagirathi	245
		RGN-13	145			Kranti	180
5	Ni4F11	Vasundhara	150	20	BrgMS301	EJ-20	195
6	BRMS-006	Pusa Mustard-28	135	21	BrgMS691	EJ-17	315
		Pusa Bahar	125	22	BrgMS175	Vasundhara	260
7	BrgMS457	PBR-210	150	23	BrgMS566	RH-406	230
8	MR52a	Pusa Bahar	145	24	BrgMS90	PBR-97	300
9	BrgMS713	EJ-20	165	25	BrgMS519	Varuna	235
10	SJ0502	Pusa Mustard-24	255	26	BrgMS64	Sarama	390
11	EJU5	Vaibhav	160	27	BrgMS360	Urvashi	280
		Pusa Jaikisan	155	28	BrgMS841	Sitara Singar	225
12	Ni2A02	JM-3	133			Ashirwad	200
13	cnu_m587a	JM-3	180	29	BrgMS89	RGN-13	265
14	Ol10B07	LET-21	145	30	BrgMS2996	Bhagirathi	210
15	BrgMS409	Urvashi	345	31	BrgMS166	Laxmi	310

## Discussion

Evaluation of genetic diversity and population structure analysis has many positive implications for genetic resource conservation and for developing an effective breeding program. The potential of identifying a superior genotype increases with the proper estimation of genetic diversity in a given population set. In the present study, 200 SSR markers distributed over all the eighteen chromosomes of Indian mustard were used for genotyping of 87 Indian mustard varieties. The advantage of using molecular markers distributed throughout the entire genome of a crop ensures that equal chances of representation is given to all the regions of the genome, thus avoiding inaccurate estimates of the genetic similarities/dissimilarities among the individuals [25]. A total of 174 SSR primer pairs amplified polymorphic products with 3.17 average allele number and 0.39 as mean PIC value. In a similar study, a lesser average number of alleles (2.37) and lower mean PIC value (0.32) than the present study when they analyzed genetic diversity in 23 Indian mustard genotypes using 16 SSR markers [6]. In another study, a lower average PIC value (0.32) was also reported when they genotyped 165 inbred lines of *B*. *oleracea* var. *botrytis* using 43 SSRs, inferring presence of narrow genetic diversity in the genotype panel [26]. Genetic diversity parameters depend upon the origin of genotypes under study, whether they are inter-related or not and the type of molecular marker used. On the contrary, higher average number of alleles (3.57) and average PIC value (0.48) per SSR locus were reported when 95 germplasm accessions of *B*. *juncea* were characterized using 44 SSR markers in a similar study [27]. A lower mean PIC value and gene diversity value obtained in the present study indicated the presence of lower level of genetic diversity among the Indian mustard varieties.

UNJ-dendrogram divided all the 87 Indian mustard varieties into two major clusters. Structure analysis also grouped the present set of varieties into two subpopulations, which is in concurrence with neighbor joining grouping method. Though few mismatches in varieties belonging to subpopulation I and cluster I and also in varieties belonging to subpopulation II and cluster II were observed, notably varieties Prakash, RH 30, Vasundhara, Rohini, NRCHB 101, RCC 4 and HYT 33 of subpopulation I were grouped into cluster II, similarly varieties Sitara Singar and PBR 210 of subpopulation II were grouped into cluster I. We observed that out of nine varieties expressing mismatches in two grouping methods, eight had an admixture ranging between 30–50% from other subpopulation, which may be one of the reasons of such mismatches. In an earlier investigation, 31 Indian mustard varieties had been grouped into five different clusters on the basis of multivariate analysis following Euclidean distance and UPGMA method [7]. In a similar study, population structure analysis had been carried out to determine the extent of genetic variation among 58 leafy mustard *(B*. *juncea* var. *rugosa*) germplasm lines using 159 SSRs [10], which classified them into four subpopulations. Population structure had also been determined in 67 *B*. *carinata* (Ethiopian mustard) germplasm lines using SSR markers and three subpopulations were obtained [12].

Selection of diverse parents in a hybridization programme is the key for creating more transgressive segregants in early generations and providing better scope for selection of desired recombinants. Clustering helps in grouping genotypes into diverse groups, but it did not speak about genetic makeup of genotype. Structure analysis reveals the extent of admixture from other subpopulations, hence provides right information for selection of parents from diverse groups. Parents for hybridization should be representative of diverse gene pools and at the same time should have minimum admixture from other subpopulations. Varieties Basanti, Sitara Singar, Pusa Karishma, RLM 619 of cluster II and Varuna, Urvashi, Pusa Mustard 25 (NPJ 112) and PBR 210 of cluster I expressed more distinctness from other varieties of their respective group, as depicted by more edge length (Fig 1). These representative varieties from each cluster may be suitable for hybridization programme to generate superior transgressive segregants. It is however not clear that whether varieties having more distinctness (more edge length) with high extent of admixture shall be appropriate for hybridization or variety with nil/least admixture from other subpopulation shall be the right choice for hybridization programme. In our case, we selected the parents with least admixture presuming more segregation in early crosses involving them. Representative varieties from cluster I; Basanti, Sitara Singar, Pusa Karishma, RLM 619 and of cluster II; Varuna, Urvashi and Pusa Mustard 25 had significant admixture from other subpopulation, hence may not be the right choice for recombination breeding within present set of varieties. Varieties with least admixture; Bhagirathi, Krishna, Pusa Mustard 29, RH 781, Kranti, Pusa Mustard 27, RLC 2, Sanjucta Asech and BR 40 of cluster I and PBR 210, Pusa Mustard 24, Pusa Mustard 25, Urvashi, NDRE 4, Pusa Jaikisan, Varuna, JM 2 of cluster 2 are recommended as parents for hybridization programme.

Pedigree, geographical diversity and trait advantage have been the criterion for selection of parents for hybridization in applied breeding since the historical times, but with the advent of molecular markers, diversity at molecular level has also been given more consideration. Reasons for narrow genetic base of *B*. *juncea* lie in the fact of its restricted geographical diversity. Cultivation of this species remained restricted largely to northern states of India, though it has shown promise in Australia and Canada. Another reason is that its large-scale cultivation in India is of recent origin. Though, as a species, *B*. *juncea* is supposed to be in existence for about 2500 years, however large-scale cultivation started only 100 years back when *B*. *juncea* due to its inherent tolerance against abiotic and biotic stresses, replaced that time prevalent *B*. *campestris* var brown sarson [15]. *B*. *juncea* (AABB) being amphidiploids expressed better tolerance to prevalent diseases and insect-pests. Genetic diversity evaluation had been carried out in *B*. *juncea* varieties on morphological basis and distinctness in varieties developed in eastern and western states than that of the northern states was obtained. We in the present study compared the diversity at molecular level among varieties developed in different states. Present set of 87 varieties was developed at 25 different centres/locations. We observed diversity in varieties of same centre which have developed more than two varieties, as witnessed by grouping into different subpopulations of varieties developed at the same centre. Pedigree-wise analysis of varieties revealed that Varuna was used as a parent in 21 varieties of which varieties; Rohini, Kranti, Pusa Jaikisan and Krishna are direct selection from Varuna, while in other 17 varieties; Basanti, RGN 13, Shivalik, Bhagirathi, GM 3, Pusa Jagannath, JM 2, JM 3, Maya, Pusa Bahar, Pusa Bold, RCC 4, Urvashi, Vardan, Vaibhav, PBR 97 and Sarama, it was one of the parents. These derivatives of Varuna namely Krishna, Pusa Bold, Pusa Jagannath, Pusa Jaikisan and Kranti were also involved as parent in 16 varieties of *B*. *juncea*. Considering direct and indirect role of Varuna in Indian national breeding programme, it contributed in parentage of 38 varieties in the present set only, which is again a major reason of narrow genetic base of *B*. *juncea*.

Among the present set of varieties, most similar varieties on the basis of SSR marker variation were Patan Mustard 67 and PCR 7; Ashirwad and Aravali; RH 406 and RH 749; Krishna and Kranti. Both the varieties PM 67 and PCR 7 were derived from germplasm collected from Gujarat, hence are expected to share common gene pool. Ashirwad and Aravali had Krishna as common parent in their pedigree. RH 406 and RH 749 both were developed at same centre, though they did not share common parents in their pedigree, however, sharing of common gene pool from germplasm is expected. Krishna being a direct selection from Kranti, hence remained as a closely similar variety. Out of 21 varieties having Varuna in their pedigree, six varieties; Vardan, Vaibhav, RL 1359, Pusa Jaikisan, Urvashi and JM 2 remained in subpopulation II, while 15 varieties acquired sufficient variation to move to subpopulation I. It was observed in structure analysis that varieties having acquired admixture from other subpopulation turned to be more distinct from other varieties of the same subpopulation. Varieties; Basanti, RLM 619, Sitara Singar, Urvashi and Pusa Karishma having 30–50% admixture expressed more distinctness from other varieties of the same group. However, the other varieties with similar extent of admixture remained similar to the other varieties of the group, so it may be inferred that distinctness depends upon the alleles introgressed and not on the extent of admixture. Thus, the earlier hypothesis that the repeated use of few widely adapted cultivars as parents in hybridization lead to narrow genetic base and yield stagnation in Indian mustard [15] has been proved in this study at molecular level using informative SSR markers.

In the present study, AMOVA results inferred the presence of significant variations among the varieties (98%) than that between subpopulations genetic variation (2%). Higher levels of variation within individuals than among subpopulations were also reported while evaluating population structure of leafy mustard (*B*. *juncea* var. *rugosa*) [10] and Ethiopian mustard (*B*. *carinata*) [12], respectively.

To establish the newness of a crop genotype or a variety, DUS (distinctness, uniformity and stability) test involves almost two years testing in field along with the reference varieties, which is quite cumbersome and time consuming [28]. On the other hand, use of molecular markers for this purpose advocates a rapid, robust, less time consuming and more reliable approach for varietal identification. In recent years, fingerprinting of Indian mustard varieties using DNA-based molecular markers is of paramount significance for unambiguous and fast identification of morphologically similar looking varieties which could prevent the disputes of varietal ownership [29]. In the present studies, unique alleles were identified that can serve as DNA fingerprints to distinguish a particular variety from other varieties. A total of 31 SSRs produced 36 unique alleles for 27 Indian mustard varieties, which can be successfully deployed as molecular tags or DNA fingerprints for quick identification of these varieties.

## Conclusion

The present study constitutes the first attempt to develop understanding about the genetic variability and development of unique DNA-fingerprints for Indian mustard varieties using SSR markers. Both cluster analysis and population structure analysis divided all the cultivars into two major groups. SSR marker variation and pedigree analysis of released varieties expressed narrow genetic base. Further, we suggest interspecific hybridization, resynthesis of *B*. *juncea*, *de novo* derivation of *B*. *juncea* from hybridization between nonparental amphidiploids, mutation breeding and population improvement methods for broadening the genetic base of *B*. *juncea* varieties. The results of the present study can be useful in formulating breeding programs of Indian mustard as they can assist in identification of genetically diverse genotypes to be used as parents for genetic improvement of this crop. The polymorphic SSRs identified in this study would facilitate marker-assisted breeding, QTL(s) and gene mapping studies through linkage analysis and association mapping studies in Indian mustard. Further, 36 unique DNA fingerprints have been developed for 27 Indian mustard varieties, which can be used for registration under Plant Varieties and Farmers’ Rights Act-2001 for obtaining plant varietal protection and also resolving disputes in seed certification. However, due to the narrow genetic base or genetic composition similarities of Indian mustard varieties, it has been concluded that more comprehensive set of SSR markers is required to characterize these varieties to develop unique DNA fingerprints for all of them.
